# Increased Patient Empowerment Is Associated with Improvement in Anxiety and Depression Symptoms in Type 2 Diabetes Mellitus: Findings from the INDICA Study

**DOI:** 10.3390/ijerph19084818

**Published:** 2022-04-15

**Authors:** Andrea Duarte-Díaz, Himar González-Pacheco, Amado Rivero-Santana, Yolanda Ramallo-Fariña, Lilisbeth Perestelo-Pérez, Yolanda Álvarez-Pérez, Wenceslao Peñate, Carme Carrion, Pedro Serrano-Aguilar, on behalf of the INDICA Team

**Affiliations:** 1 Canary Islands Health Research Institute Foundation (FIISC), 38109 El Rosario, Spain; andrea.duartediaz@sescs.es (A.D.-D.); himar.gonzalezpacheco@sescs.es (H.G.-P.); amado.riverosantana@sescs.es (A.R.-S.); yolanda.ramallofarina@sescs.es (Y.R.-F.); yolanda.alvarezperez@sescs.es (Y.Á.-P.); 2 Department of Clinical Psychology, Psychobiology and Methodology, Universidad de La Laguna (ULL), 38200 San Cristóbal de La Laguna, Spain; wpenate@ull.edu.es; 3 Research Network on Health Services in Chronic Diseases (REDISSEC), 38109 El Rosario, Spain; pseragu@gobiernodecanarias.org; 4 Network for Research on Chronicity, Primary Care, and Health Promotion (RICAPPS), 38109 El Rosario, Spain; 5 Evaluation Unit (SESCS), Canary Islands Health Service (SCS), 38109 El Rosario, Spain; 6 eHealth Lab Research Group, School of Health Sciences, Universitat Oberta de Catalunya (UOC), 08035 Barcelona, Spain; mcarrionr@uoc.edu

**Keywords:** empowerment, patient-centered care, type 2 diabetes mellitus, anxiety, depression

## Abstract

Introduction. In cross-sectional analyses, higher levels of patient empowerment have been related to lower symptoms of anxiety and depression. The aims of this study are: (1) to assess if patient empowerment predicts anxiety and depression symptoms after 12 and 24 months among patients with type 2 diabetes mellitus, and (2) to analyze whether a change in patient empowerment is associated with a change in anxiety and depression level. Methods. This is a secondary analysis of the INDICA study, a 24 month-long, multi-arm randomized controlled trial. Patient empowerment (DES-SF), depression (BDI-II), and state-anxiety (STAI-S) were assessed at the baseline (pre-intervention) and after 12 and 24 months. Multilevel mixed linear models with a random intercept were performed to correct for our clustered data. Results. The multilevel regression models showed that the baseline empowerment did not significantly predict anxiety and depression after 12 and 24 months. However, a higher increase in patient empowerment was significantly associated with reductions of anxiety (*p* < 0.001) and depression levels (*p* < 0.001). This association was not significantly different between the two follow-ups. Conclusion. This study contributes to the knowledge on how to reduce affective symptoms in patients with uncomplicated T2DM through comprehensive patient-centered interventions, and it highlights patient empowerment as a significant contributor.

## 1. Introduction

Diabetes mellitus (DM) is a chronic metabolic disorder characterized by the presence of hyperglycemia due to an impairment of insulin secretion, defective insulin action, or both [1]. DM constitutes a growing public health problem. Worldwide DM prevalence in adults from 20 to 79 years old in 2021 was estimated to be 10.5% (536.6 million people), and it is expected to rise to 12.2% (783.2 million people) in 2045 [2]. Type 2 diabetes mellitus (T2DM) represents over 90% of DM cases around the world [3], and the continued rise to 2045 is projected to be greater for T2DM patients [2]. In Spain, according to a nation-wide population-based study, the incidence of T2DM was recently estimated to be 11.6 cases/1000 person-year [4], and the prevalence rate has been found to be higher in the Canary Islands than in the rest of the country [5].

Depression is a very common comorbidity in patients with T2DM. Almost one in four European adults suffer from different severity levels of depressive disorders [6], with only slightly lower prevalence rates in Spain [7]. Additionally, an international study has shown that people with T2DM have a higher prevalence of anxiety disorders, especially women, with diabetes-related complications, increased duration of the illness, and poorer glycemic control [8]. Affective and emotional disorders in patients with T2DM have been associated with non-adherence to medication [9], diet, and physical activity [10,11] and also with poorer quality of life [12]. Despite this evidence, recognizing and addressing psychological symptoms in T2DM healthcare management remain challenging. Identifying and treating mental health comorbidities among patients with diabetes must be a priority [13], and specific strategies should be considered in effective diabetes management programs to reduce its impact on health outcomes [14].

According to the American Diabetes Association and the European Association for the Study of Diabetes, psychological care should be integrated with a collaborative, patient-centered approach and provided to all people with diabetes to optimize health outcomes and quality of life [15,16]. In their position statement about psychological care for people with diabetes, patient-centered care was defined as an essential factor to promote optical medical outcomes and psychological well-being [17]. Patient-centered care refers to a holistic approach to deliver care in a respectful and individualized way, allowing negotiation of care and offering choices through a therapeutic relationship where patients are empowered to be actively involved in health decisions [18]. In a classical conceptualization, Funnel et al. defined patient empowerment as a conceptual shift in the relationship between patients and providers, where patients are no longer consumers of services but active partners in the provision of their diabetes care [19]. From this point of view, empowered patients are those having the knowledge, skills, attitudes, and self-awareness necessary to influence their own behavior in order to improve the quality of their lives [19]. However, multiple different definitions of patient empowerment are available in the literature [20,21,22], and its boundaries with other theoretically-related constructs, such as self-efficacy, enablement, or patient activation, still present many ambiguities [23]. This makes it difficult to reach a consensus about the conceptualization and measurement of patient empowerment, but it has been suggested to be a wide-ranging and multidimensional concept [24], which could be potentially measurable using those related indicators [25].

In cross-sectional analyses, higher levels of patient empowerment have been related to lower symptoms of anxiety and depression in patients with T2DM [26,27,28]. Additionally, there is some evidence suggesting that interventions aimed to promote empowerment and patient activation can alleviate affective symptoms in different settings such as hemodialysis [29] or chronic diseases, including cancer, chronic respiratory diseases, and diabetes [30].

There is an increasing call for a move toward a more patient-centered care in the field of diabetes [16]. Furthermore, for optimal disease management, it is crucial that T2DM healthcare management includes strategies for the early detection and treatment of affective symptoms. Since patient empowerment is an important contributor to patient-centeredness [31], it could be considered when designing interventions from a comprehensive approach while integrating mental health management into overall diabetes care. However, a better understanding of the complex relationship between patient empowerment and affective symptoms is needed first.

In this sense, for the current analysis, we used data from the INDICA study, a multi-arm randomized controlled trial assessing the effectiveness and cost-effectiveness of different multicomponent interventions based on the conceptual framework of behavioral change and patient-centered care [32]. This study, carried out with a large sample of TSDM patients during two years, showed significant improvements in HbA1c levels (especially in those with worse control at baseline), diastolic and systolic pressure [32], adherence to the Mediterranean diet, smoking cessation, as well as in empowerment and other psychological outcomes [33]. Here, we analyzed whether patient empowerment significantly predicts anxiety and depression after 12 and 24 months among patients with T2DM and whether change in patient empowerment relates to change in affective symptoms. We hypothesized that baseline patient empowerment would predict anxiety and depression symptoms after 12 and 24 months and that increased patient empowerment would be significantly associated with improvement in both anxious and depressive symptoms.

## 2. Materials and Methods

### 2.1. Study Design

The INDICA study was a multi-arm cluster randomized controlled trial conducted in the Canary Islands, Spain (ClinicalTrials identifier NCT01657227). A detailed study description and its main results are described elsewhere [32,33,34]. The scientific and ethics committee of the University Hospital Nuestra Señora de la Candelaria (ID: EPA-07/10) approved the study protocol. The study was performed in accordance with good clinical practice standards, applicable local regulatory requirements, and the Declaration of Helsinki.

### 2.2. Setting and Recruitment

The unit of recruitment in the INDICA study was the family care unit (FCU), comprising a family physician and a nurse from primary healthcare centers (PHC) in Tenerife, Gran Canaria, Lanzarote, and La Palma. First, PHC (clusters) with at least eight FCU were recruited. Then, FCUs were randomly selected from all those consenting to participate in each PHC. After that, all potential eligible patients in each selected FCU were identified through electronic clinical records and invited to participate. Finally, patients were randomly selected from all those fulfilling the inclusion criteria and providing informed consent.

### 2.3. Participants

The INDICA study included adult patients with T2DM, without severe comorbidities, and who were diagnosed at least one year before enrolment. All participants provided written informed consent before enrollment. The full inclusion and exclusion criteria are shown in Table 1.

### 2.4. Interventions

In the INDICA study, participants were randomly allocated by clusters to usual care or to one of three multicomponent interventions of knowledge transfer and behavior modification: one aimed directly at patients (PTI), one aimed at primary care healthcare professionals (PFI), and one offering both interventions simultaneously (CBI). The intervention aimed at patients included: (1) a set of eight group sessions, during the 2 years of intervention, aimed to empower patients in self-control and monitoring; (2) monitoring of physical activity, diet, drug adherence, mood, blood pressure, and blood glucose; and (3) continuous, personalized feedback by semi-automated mobile phone messages. The intervention for healthcare professionals included: (1) an educational and interactive group program of two sessions, three months apart, with updated clinical management information and training on how to provide patient-centered care; (2) an automated decision aid tool based on a clinical practice guide for T2DM; and (3) monthly computerized graphic feedback on outcome indicators for all patients with T2DM.

### 2.5. Measures

All measures were administered at the baseline (pre-intervention) and after 12 and 24 months of intervention.

#### 2.5.1. Patient Empowerment

The Diabetes Empowerment Scale-Short Form (DES-SF) [35] was used to assess empowerment levels. It is an eight-item short form of the original Diabetes Empowerment Scale (DES) [36], specifically designed to allow for a brief overall assessment of diabetes-related psychosocial self-efficacy, including three aspects: (1) the management of the psychological aspects of diabetes; (2) the assessment of dissatisfaction and readiness to change; and (3) setting and achieving diabetes goals. The eight items of the DES-SF are rated on a 5-point Likert scale, ranging from “strongly disagree” to “strongly agree”. The total score ranges from 8 to 40, and higher scores indicate stronger levels of patient empowerment. The DES-SF has shown good internal consistency (Cronbach’s alpha coefficient of 0.83) and stability over time (r = 0.532; *p* = 0.009) [37]. The DES-SF has been translated and adapted for Spanish-speaking older adults with chronic diseases by replacing the word “diabetes” with “health” to cover all kinds of health conditions [38]. This adaptation has shown good internal consistency (Cronbach’s alpha of 0.89) and convergent validity with the General Self Efficacy Scale (r = 0.77) [38].

#### 2.5.2. Depression

Depression level was assessed by the Beck Depression Inventory-II (BDI-II) [39], a 21-item self-reported instrument designed to measure and quantify the severity of depressive symptoms. The questionnaire consists of 21 items, each rated on a 4-point Likert scale (from 0 to 3). The minimum and maximum scores are 0 and 63, where the higher scores indicate greater severity of depressive symptoms. More specifically, a score of 0–13 indicates minimal or no depression; 14–19 mild depression; 20–28 moderate depression; and a score of 29–63 indicates severe depression. The BDI-II is a reliable and well-validated measure. Studies on its psychometric properties have reported an average Cronbach’s alpha around 0.9 and good-to-excellent retest reliability coefficients (range 0.73 to 0.96) [40]. The Spanish adaptation [41] has also shown good internal consistency in general [42], medical [43], and psychiatric samples [41] (Cronbach’s alpha of 0.87, 0.92, and 0.89 respectively).

#### 2.5.3. Anxiety

The Spanish adaptation [44] of the State-Trait Anxiety Inventory (STAI) [45] was used to determine anxiety levels of T2DM patients. It is a psychological self-reported 40-item inventory. The first 20 items measure the state of anxiety (STAI-S), and the second 20 items assess trait anxiety (STAI-T). In the Spanish version, each item is rated on a 4-point Likert scale from 0 (not at all/almost never) to 3 (very much so/almost always). The total score for each subscale is obtained from the sum of the individual items, considering the inversion of the negative ones. Therefore, the total score oscillates between 0 and 60, with higher scores reflecting greater anxiety. Both subscales have shown good reliability (Cronbach’s alpha 0.90 and 0.94 for trait and state subscales, respectively) [46]. Here, we used only the state subscale, which measures a transient emotional state characterized by perceived subjective feelings of tension and apprehension.

#### 2.5.4. Control Variables

To control potential confounding, all multivariate analyses were adjusted for age and time since diagnosis.

### 2.6. Statistical Analyses

Statistical analyses were performed using STATA 15.0 software. The main characteristics of the sample and the study variables were described using frequencies and percentages for categorical variables and mean and standard deviation for continuous variables.

First, the hypotheses of a linear association between baseline empowerment scores and anxiety and depression after 12 and 24 months, respectively, were assessed through multilevel mixed models. We conducted separate models for each dependent variable, state-anxiety (STAI-S) and depression (BDI-II), each one separately for 12 and 24 months. The first level included patients’ variables (baseline value of the dependent variable, intervention group, age, time since diagnosis, and the interaction between the intervention group and the baseline empowerment scores) and the second level corresponded to the PHC in which patients are grouped.

Next, the hypotheses of linear association between increased patient empowerment and the reduction of anxiety and depression symptoms over time were also assessed through multilevel mixed models. Separate multilevel mixed models were conducted for each dependent variable: change in state-anxiety (STAI-S) and change in depressive symptoms (BDI-II). First level variables are those corresponding to change in anxious and depressive symptoms between the baseline and the 12- and 24-months follow-ups (repeated time measures). The second level includes patients’ variables (intervention group, age, and time since diagnosis) and two interaction terms: (1) between period of time (time between baseline and 12 and 24-months) and change in patient empowerment, and (2) between intervention group and change in patient empowerment. The third level variable was the PHC in which patients were grouped.

Multilevel mixed models were performed to correct for our clustered data. The effect that identified the intervention arm was considered fixed for the different PHC, while the intercept was considered random. The intraclass correlation (ICC) was obtained for each model for the PHC and by patient according to their PHC. All the analyses were performed on an intention-to-treat basis. Missing values were treated by means of multiple imputation procedures [47] with results based on 100 imputed datasets (missing values from the follow-up visits were imputed). More details on the imputation process can be found in previous publications [33,34]. A threshold of 0.05 was used to define the statistical significance of the tests.

## 3. Results

### 3.1. Study Participants

A total of 2334 patients with uncomplicated T2DM, from 32 PHC, participated in this study. The mean age was 55.70 ± 7.1 years, and 51.9% were females. Concerning education level, 62.5% had primary or lower education, while 37.5% had secondary or higher education. Mean time since diagnosis was 8.50 ± 6.51 years, and 75.3% of the participants had HbA1c levels within the accepted therapeutic goal (<8%). At baseline, patients were not highly anxious or depressed. Table 2 describes the sociodemographic and clinical data of the study sample at the baseline.

**Figure 1:**
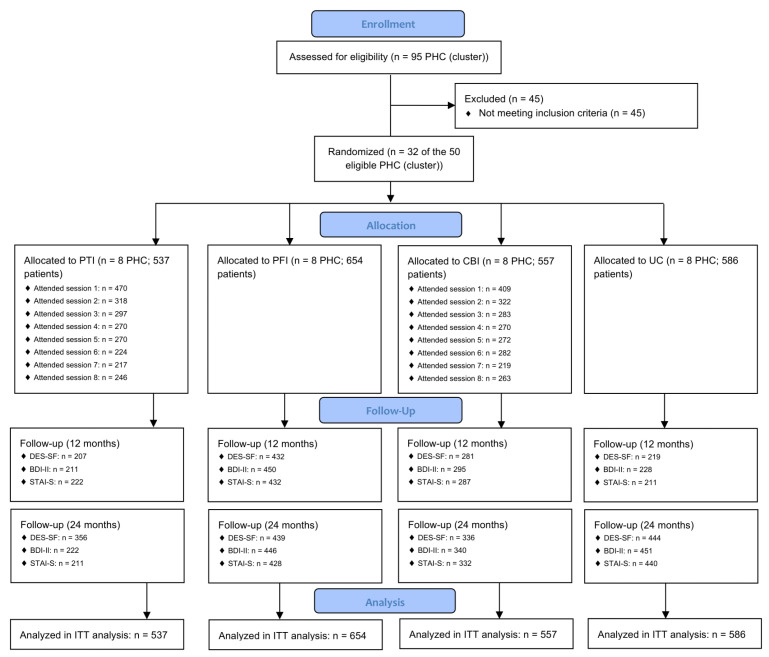
Flowchart illustrating the progress of participants throughout the trial. BDI-II: Beck Depression Inventory-II; DES-SF: Diabetes Empowerment Scale-Short Form; ITT: intention-to-treat; STAI-S: State subscale of the Trait Anxiety Inventory; PHC: Primary Healthcare Center; UC: usual care.

The flowchart with the cluster randomization of patients for each intervention, the attendance rate at educational/training sessions of patients and professionals, and the number of questionnaires received for each follow-up assessment is shown in Figure 1.

### 3.2. Patient Empowerment as a Predictor of Anxiety

Contrary to our hypotheses, baseline patient empowerment did not significantly predict anxiety scores, neither after 12 (*p* = 0.219) nor 24 months (*p* = 0.115) (Table 3). The interaction between the intervention group and empowerment was not significant; that is, the association between baseline empowerment and anxiety at the follow up did not significantly differ between experimental groups (the interaction term was excluded from the models shown in Table 3).

Baseline anxiety significantly predicted anxiety scores at 12 and 24 months (Table 3). At 12 months, this association was significantly weaker in the mixed intervention group (B = 0.17, *p* = 0.001) compared to the usual care group (B = 0.41, *p* < 0.001) (*p* = 0.005 for the between-group difference). At 24 months, this association was not significantly different between the four study groups (data not shown).

### 3.3. Patient Empowerment as a Predictor of Depressive Symptoms

Contrary to what was expected, baseline patient empowerment did not significantly predict depression scores after 12 (*p* = 0.226) or 24 months (*p* = 0.108) (Table 4). The interaction between the intervention group and empowerment was not significant, and it was excluded from the models shown in Table 4.

Baseline depression significantly predicted depression scores at 12 and 24 months (Table 4). At 12 months, this association was significantly weaker in the professional (B = 0.30, *p* < 0.001) and mixed (B = 0.24, *p* < 0.001) intervention groups compared to the usual care group (B = 0.57, *p* < 0.001) (*p* < 0.001 for the between-group differences). At 24 months, this association was not significantly different between the four study groups (data not shown).

### 3.4. Change in Patient Empowerment and its Association with Change in Anxiety Symptoms

The multilevel regression model (Table 5) showed that a higher increase in patient empowerment was significantly associated with greater anxiety reduction (B = −0.23; *p* < 0.001), and this effect was not significantly different between the two follow-ups (interaction time × DES-SF non-significant, *p* = 0.646). The interaction term between the intervention group and the change in patient empowerment was not significant, so it was excluded from the model. The ICC was small at the PHC level but broad at the patient level, accounting for considerable variations among patients

### 3.5. Change in Patient Empowerment and its Association with Change in Depressive Symptoms

Just as for anxiety symptoms, the multilevel regression model (Table 6) showed that a higher increase in patient empowerment was significantly associated with greater depression reduction (B = −0.12, *p* < 0.001), without a significant interaction time × DES-SF (*p* = 0.381). The interaction term between the intervention group and change in patient empowerment was not significant and was excluded from the model.

## 4. Discussion

The mechanism underlying how patient empowerment is linked to affective outcomes in T2DM patients has not been clearly elucidated. Contrary to what was expected, the findings of this secondary analysis of the INDICA study suggest that patient empowerment does not predict anxiety and depression scores after 12 or 24 months. However, the results do support our a priori hypothesis that increased patient empowerment would be associated with improvements in both anxiety and depression symptoms.

The results of this study extend previous findings that empowering patients to become more actively involved in their care could lead to better mental wellbeing. In a large prospective cohort study with moderate-to-severe depression, higher baseline patient activation was found to be predictive of more reduction in depressive symptoms and a larger percentage of remission and response to treatment over one year [48]. Previous evidence in the field of diabetes has also contributed to shed light on this association. In this respect, different studies have found that increased self-efficacy or patient activation were significantly related to improvements in depressive mood [49,50,51]. Similarly, intervention programs that include empowerment as a primary goal may improve psychological outcomes as well as enhance quality of life [52,53]. Hernández-Jiménez et al. assessed the effectiveness of a comprehensive care program based on empowerment techniques to achieve metabolic goals and improve other diabetes-related complications, such as anxiety and depression symptoms [52]. After the three-month intervention phase, the percentage of T2DM patients suffering from anxiety and depression significantly decreased, and the scores remained low at the annual follow-ups [52]. Likewise, Cheng et al. tested the application of a collaborative empowerment-based self-management intervention specifically aimed towards patients with poorly controlled T2DM [53]. As they concluded, both emotional and regimen-related distress were responsive to the intervention and were considerably reduced at three months post-intervention [53]. Similar results have also been found with our INDICA interventions, which included empowerment techniques based on the conceptual framework of patient-centered care [34]. After 12 months, and compared to usual care, the intervention aimed at professionals (PFI) and the one targeting patients and professionals jointly (CBI), showed statistically significant effects on patient empowerment scores and also on the reduction of anxious and depressive symptoms [32]. These effects were not observed with the intervention targeting exclusively patients (PTI) and could be interpreted as the result of providing better patient-centered care, which also underlines the importance of training healthcare professionals on how to move toward patient-centeredness and promote patient empowerment.

These findings are consistent with Bandura’s postulates. According to his Social Cognitive Theory [54], self-efficacy, one of the main indicators of patient empowerment, plays an essential role in the self-regulation of affective states [55,56]. Since they manifest more doubts about their capabilities, patients with low self-efficacy may believe they are inefficient at dealing with the daily demands of diabetes, which in turn results in more stress, anxiety or depression. As Bandura himself noted, *“the inability to influence events and social conditions that significantly affect one’s life can give rise to feelings of futility and despondency as well as anxiety”* (p. 153) [55].

In light of our results and previous evidence, it can be assumed that empowering patients through enhancing patient activation and promoting self-efficacy can have a substantial impact on T2DM patients’ mental health. Moreover, this positive effect on psychological outcomes could also contribute to better glycemic outcomes and minimize diabetes-related complications. It is widely known that optimum adherence to self-care strategies is fundamental to effectively managing diabetes. Diabetes-related emotional distress in patients with T2DM is associated with poorer treatment adherence and glycemic control, and these relationships have been found to be partially mediated through perceived control [57]. Baseline data drawn from a randomized controlled trial showed that diabetes-related distress, diabetes empowerment, and depression are statistically significant predictors of mastery, while highlighting that diabetes empowerment is positively associated with diabetes management [58]. To date, many studies have focused on the association between affective symptoms and self-care behaviors in diabetic patients [10,59,60,61,62,63]. In this respect, affective symptoms have been linked to medication non-adherence [60,63] and lower adherence to diet [10,62] and exercise recommendations [10]. The reason why depressive symptoms are associated with increased odds of suboptimal diabetes self-care probably lies in the presence of anhedonia, one of its main symptoms. Anhedonia is defined as a lack of enjoyment from, engagement in, or energy for life’s experiences, with deficits in the capacity to feel pleasure or take interest in things [64]. Accordingly, people with T2DM and comorbid depressive symptoms may be less likely to experience satisfaction or pleasure from achieving glycemic control through the maintenance of adequate self-care behaviors [65,66].

The strengths and limitations of the original INDICA study have been reported in detail elsewhere [32,33]. In brief, the trial had a large sample size and a robust cluster randomized design, with reasonably well-matched participants in the control and intervention groups, and the study was successful in minimizing contamination between practices. Importantly, the intervention was designed for consistent reproducibility of training and had a relatively low up-front training investment, enabling implementation across other sites. All educators participating in the intervention were fully trained and quality assured, ensuring generalizability of the findings out of the research setting. Statistical analyses for this study were undertaken using intention-to-treat analysis and robust imputation techniques, minimizing bias in the reported findings. This secondary study had some limitations. The main one is the use of the short form of the DES, which may reduce scores’ variability, obscuring to some extent its associations with the dependent variables. Second, our study focused on patients with uncomplicated T2DM and low levels of anxiety/depression, and therefore our findings are not generalizable to patients who have already developed long-term diabetes complications, which in turn are related to severe forms of anxiety and depression [67]. Additionally, it has been recently shown that diabetes-specific emotional distress probably has a greater impact on glycemic control than depression [27,53]. The construct of diabetes distress is linked to specific health threats produced by the disease, and therefore it is more content-specific and refers to a broader affective experience than clinical depression [68]. It is not a psychopathological condition but an expected reaction to health-related stressors (e.g., starting insulin, acute and long-term complications). There is considerable debate about the independent influence of depression and diabetes distress on disease self-management and glucose control [58,61,62,69].

This study adds empirical evidence about the role of patient empowerment as an important mental health promotion strategy that may be useful to include when designing interventions for T2DM. The results of this secondary analysis of the INDICA study contribute to the knowledge on how to reduce affective symptoms through comprehensive patient-centered interventions and highlight patient empowerment as a significant contributor.

## 5. Conclusions

In conclusion, this study showed that positive changes in patient empowerment are related to improvements in both anxiety and depression symptoms in patients with T2DM.

## Figures and Tables

**Table 1:** Inclusion and exclusion criteria.

Inclusion Criteria	Exclusion Criteria
Patients with T2DM diagnosed at least one year prior to study enrolment. 18–65 years of age. Formal consent to participate in the study. Regular use of mobile phone.	Chronic kidney disease ≥ stage 3b, as defined by the National Kidney Foundation’s Kidney Disease Outcomes and Quality Improvement Initiative urinary albumin to creatinine ratio ≥300 mg/g, and/or urinary protein excretion ≥300 mg/24 h. Acute coronary syndrome (documented angina or myocardial infarction) or stroke in the last 6 months or class III or IV heart failure, according to the New York Heart Association. Proliferative diabetic retinopathy or clinically significant diabetic macular edema requiring previous treatment with retinal photocoagulation, vitrectomy, or intravitreal injections of anti-vascular endothelial growth factor or triamcinolone acetonide 6 months prior to study inclusion. Uncorrected severe hearing or visual impairment or corrected visual acuity ≤20/40 by any cause. Diabetic foot with ulcers ≥2 according to the Wagner scale. Liver cirrhosis. Cancer unless disease free 5 years after diagnosis. Other terminal illnesses. Intellectual disability, dementia, or psychotic diseases. Active substance abuse. Pregnancy. Insufficient Spanish language skills. Physical disability limiting participation in group education activities. Concurrent participation in another clinical trial or any other investigational study.

T2DM: type 2 diabetes mellitus.

**Table 2:** Sociodemographic and clinical characteristics.

Characteristics	*n* (%) or Mean (SD)
Age (years), mean (SD)	55.70 (7.1)
Gender, *n* (%) Male Female	1.122 (48.1) 1.212 (51.9)
Education level, *n* (%) Primary or lower Secondary or higher	1.458 (62.5) 876 (37.5)
Living alone, *n* (%) Yes No	209 (8) 2.125 (91)
Marital status, *n* (%) With partner Without partner	1.821 (78) 513 (23)
Employment status, *n* (%) Active Non-active	1.367 (58.6) 967 (41.4)
Monthly income, *n* (%) <€500 ≥€500	1.742 (74.6) 592 (25.4)
Years since diagnosis, mean (SD)	8.50 (6.51)
HbA1c, *n* (%) <8% ≥8%	1.758 (75.3) 576 (24.7)
HbA1c, mean (SD)	7.28 (1.47)
BMI, *n* (%) Normal-underweight (<25) Overweight (<30) Class I obesity (<35) Class II obesity (<40) Class III obesity (≥40)	199 (8.6) 726 (31.1) 794 (34) 402 (17.2) 213 (9.1)
BMI, mean (SD)	32.07 (5.91)
Number of comorbidities, mean (SD)	2.75 (1.85)
WHI, mean (SD)	0.98 (0.07)
Creatine, mean (SD)	0.80 (0.18)
Triglycerides, mean (SD)	162.53 (104.35)
Patient empowerment (DES-SF), mean (SD)	26.58 (9.17)
Depression (BDI-II), mean (SD)	11.26 (9.90)
Anxiety (STAI-S), mean (SD)	21.77 (13.49)

BDI-II: Beck Depression Inventory-II; BMI: body mass index; DES-SF: Diabetes Empowerment Scale-Short Form; HbA1c: glycated hemoglobin; SD: standard deviation; STAI-S: State subscale of the Trait Anxiety Inventory; WHI: Waist Hip Index.

**Table 3:** Regression model for state-anxiety (STAI-S) after 12 and 24 months.

*n* = 2334	12 Months			24 Months		
	Coefficient (CI95%)	*t*-Student	*p*-Value	Coefficient (CI95%)	*t*-Student	*p*-Value
Intervention (ref: usual care)						
Patients	−2.34 (−6.14; 1.45)	−1.21	0.226	−2.00 (−6.04; 2.04)	−0.97	0.331
Professionals	−3.49 (−7.28; 0.31)	−1.80	0.072	−0.33 (−4.38; 3.72)	−0.16	0.873
Mixed	−5.26 (−9.15; −1.36)	−2.65	0.008	−0.50 (−4.55; 3.56)	−0.24	0.811
STAI-S (baseline)	0.32 (0.27; 0.37)	13.06	0.000	0.22 (0.18; 0.27)	9.62	0.000
DES-SF (baseline)	−0.04 (−0.10; 0.02)	−1.23	0.219	−0.05 (−0.11; 0.01)	−1.58	0.115
Time since diagnosis	0.79 (−0.02; 0.17)	1.63	0.104	0.04 (−0.05; 0.12)	0.92	0.359
Age	−0.04 (−0.14; 0.05)	−0.94	0.349	−0.05 (−0.13; 0.04)	−1.11	0.266
Constant	14.72 (11.28; 18.16)	8.39	0.000	13.07 (9.59; 16.56)	7.35	0.000
	F = 34.29; *p* < 0.000; ICC PHC = 0.09			F = 18.80; *p* < 0.000; ICC PHC = 0.11		

DES-SF: Diabetes Empowerment Scale-Short Form; ICC: intra-class correlation; PHC: Primary Healthcare Center; ref: reference; STAI-S: State subscale of the Trait Anxiety Inventory.

**Table 4:** Regression model for depressive symptoms (BDI-II) after 12 and 24 months.

*n* = 2334	12 Months			24 Months		
	Coefficient (CI95%)	*t*-Student	*p*-Value	Coefficient (CI95%)	*t*-Student	*p*-Value
Intervention (ref: usual care)						
Patients	−1.98 (−4.02; 0.07)	−1.89	0.059	−0.68 (−3.02; 1.65)	−0.58	0.565
Professionals	−3.08 (−5.05; −1.11)	−3.07	0.002	0.45 (−1.88; 2.78)	0.38	0.705
Mixed	−2.94 (−5.04; −0.83)	−2.73	0.006	0.23 (−2.13; 2.59)	0.19	0.846
BDI-II (baseline)	0.40 (0.34; 0.46)	13.48	0.000	0.24 (0.20; 0.28)	12.35	0.000
DES-SF (baseline)	−0.03 (−0.8; 0.18)	−1.21	0.226	−0.03 (−0.08; 0.01)	−1.61	0.108
Time since diagnosis	0.02 (−0.04; 0.09)	0.68	0.495	0.02 (−0.03; 0.08)	0.82	0.412
Age	−0.00 (−0.07; 0.06)	−0.04	0.971	−0.01 (−0.06; 0.05)	−0.25	0.802
Constant	6.77 (4.77; 8.78)	6.63	0.000	5.00 (2.96, 7.02)	4.81	0.000
	F = 51.65; *p* < 0.000; ICC PHC = 0.04			F = 27.58; *p* < 0.000; ICC PHC = 0.09		

BDI-II: Beck Depression Inventory-II; DES-SF: Diabetes Empowerment Scale-Short Form; ICC: intra-class correlation; PHC: Primary Healthcare Center; ref: reference.

**Table 5:** Regression model for change in state-anxiety (STAI-S).

*n* = 2334	Coefficient (CI95%)	*t*-Student	*p*-Value
Intervention (ref: usual care)			
Patients	−1.39 (−4.98; 2.20)	−0.76	0.449
Professionals	−0.23 (−4.24; 3.79)	−0.11	0.912
Mixed	−3.19 (−6.98; 0.60)	−1.65	0.099
Time (change 24 month-baseline) (ref: change 12 months-baseline)	−1.64 (−2.53; −0.75)	−3.62	<0.001
DES-SF (change 24 month-baseline) (ref: change 12 months-baseline)	−0.23 (−0.30; −0.16)	−6.28	<0.001
Time × DES-SF (change 24 month-baseline) (ref: change 12 months-baseline)	0.02 (−0.06; 0.10)	0.46	0.646
Time since diagnosis	0.08 (−0.03; 0.19)	1.35	0.176
Age	0.07 (−0.03; 0.18)	1.4	0.163
Constant	−1.75 (−4.39; 0.90)	−1.3	0.195
F = 12.81; *p* < 0.001; ICC PHC = 0.04; ICC subject | PHC = 0.60			

DES-SF: Diabetes Empowerment Scale-Short Form; ICC: intra-class correlation; PHC: Primary Healthcare Center; ref: reference.

**Table 6:** Regression model for the change in depressive symptoms (BDI-II).

*n* = 2334	Coefficient (CI95%)	*t*-Student	*p*-Value
Intervention (ref: usual care)			
Patients	−0.80 (−2.85; 1.26)	−0.76	0.446
Professionals	−0.75 (−3.0; 1.49)	−0.66	0.509
Mixed	−1.21 (−3.39; 0.97)	−1.09	0.277
Time (change 24 month-baseline) (ref: change 12 months-baseline)	−1.52 (−2.08; −0.96)	−5.32	<0.001
DES-SF (change 24 month-baseline) (ref: change 12 months-baseline)	−0.12 (−0.17; −0.07)	−4.71	<0.001
Time × DES-SF (change 24 month-baseline) (ref: change 12 months-baseline)	0.02 (−0.03; 0.07)	0.88	0.381
Time since diagnosis	0.01 (−0.06; 0.08)	0.22	0.828
Age	0.03 (−0.04; 0.10)	0.77	0.444
Constant	−1.61 (−3.10; 0.12)	−2.12	0.034
F = 10.49; *p* < 0.001; ICC PHC = 0.02; ICC subject | PHC = 0.51			

DES-SF: Diabetes Empowerment Scale-Short Form; ICC: intra-class correlation; PHC: Primary Healthcare Center; ref: reference.

## Data Availability

The data presented in this study are available within the article.

## Supplementary Materials

The following supporting information can be downloaded at: https://www.mdpi.com/article/10.3390/ijerph19084818/s1.
