# Supplementary material for: Increased Patient Empowerment Is Associated with Improvement in Anxiety and Depression Symptoms in Type 2 Diabetes Mellitus: Findings from the INDICA Study
Source: Int J Environ Res Public Health. 2022 Apr 15;19(8):4818. doi: 10.3390/ijerph19084818 (PMC9028935; doi:10.3390/ijerph19084818)
Supplement: Supplementary file 1 [file ijerph-19-04818-s001.zip › ijerph-1634239-supplementary.pdf]

International Journal of
Environmental Research and Public Health

MDPI

Supplementary Materials

Collaborators: The INDICA team included the following members: Abraham Pérez de la Rosa (Canary Islands Health Research Institute Foundation, FIISC), Alicia Pareja Ríos (University Hospital of Canary Island), Ana Wägner (Insular University Hospital), Andrés Sifre Perello (Molina Orosa Hospital), Ángela Trinidad Gutiérrez Pérez (Primary Care of Gran Canaria), Antonio Cabrera de León (Ntra Sra de la Candelaria University Hospital), Antonio García Quintana (Dr. Negrín University Hospital), Armando Carrillo Domínguez (Insular University Hospital), Bernardo Eusebio Herrera Domínguez (General de La Palma Hospital), Carlos Sedeño Pérez (Primary Care of Tenerife), Carlos Ramírez Álamo (Primary Care of Gran Canaria), Carmen Daranas Aguilar (Canary Islands Health Research Institute Foundation, FIISC), Carolina Guerra Marrero (Canary Islands Health Research Institute Foundation, FIISC), Cecilia Lobos Soto (Insular University Hospital), Cristina Padrón Pérez (Canary Islands Health Research Institute Foundation, FIISC), Dácil Alvarado Martel (Dr. Negrín University Hospital), Daniel Hernández Obregón (Dr. Negrín University Hospital), Dulce N. Hernández Correa (Primary Care of Gran Canaria), Elsa Espinosa Pozuelo (Diabetes Patient' association of Tenerife), Elsa Florido Mayor (Canary Islands Health Research Institute Foundation, FIISC), Engracia Pinilla Domínguez (Ntra Sra de la Candelaria University Hospital), Fátima Herrera García (University Hospital of Canary Island), Félix Bonilla Aguiar (Dr. José Molina Hospital), Francisco Cabrera López (Insular University Hospital), Gloria Guerra de la Torre (Primary Care of Gran Canaria), Gregorio Muelas Martín (Dr. Negrín University Hospital), Guillermo Monzón Monzón (Primary Care of Gran Canaria), Héctor de la Rosa Merino (Canary Islands Health Research Institute Foundation, FIISC), Ignacio García Puente (Dr. Negrín University Hospital), Ignacio Llorente Gómez de Segura (Ntra Sra de la Candelaria University Hospital), Isabel García Calcerrada (Ntra Sra de la Candelaria University Hospital), Jacqueline Álvarez Pérez (Canary Islands Health Research Institute Foundation, FIISC), Jorge Federico Aldunate Page (Insular University Hospital), Jose Antonio García Dopico (University Hospital of Canary Island), Juan Andrés Báez Hernández (Primary Care of La Palma), Juan José Pérez Valencia (Primary Care of Tenerife), Julia Charlotte Wiebe (Dr. Negrín University Hospital), Leticia Rodríguez Rodríguez (Canary Islands Health Research Institute Foundation, FIISC), Lidia García Pérez (Canary Islands Health Research Institute Foundation, FIISC), Leopoldo Martín Martín (Hospital General de La Palma), Luis Morcillo Herrera (University Hospital of Canary Island), Marcos Estupiñán Ramírez (Canary Islands Health Service, SCS), María Inmaculada González Pérez (Ntra Sra de la Candelaria University Hospital), María Isabel Visuerte Morales (University Hospital of Canary Island), María Pino Afonso Medina (Dr. Negrín University Hospital), Margarita Roldán Ruano (Primary Care of Gran Canaria), Marta Riaño Ruiz (Insular University Hospital), Marta Tejera Santana (Dr. Negrín University Hospital), Mauro Boronat (Insular University Hospital), Mercedes Lorenzo Medina (Dr. Negrín University Hospital), Miguel Ángel García Bello (Canary Islands Health Research Institute Foundation, FIISC), Miguel Juan Mora García (Primary Care of Gran Canaria), Nayra Pérez Delgado (Ntra Sra de la Candelaria University Hospital), Pablo Pedrianez Martín (Dr. Negrín University Hospital), Pedro de Pablos- Velasco (Dr. Negrín University Hospital), Pilar Peláez Alba (La Laguna University), Rafael Valcárcel (Primary Care of Tenerife), Remedios Castro Sánchez (Primary Care of Gran Canaria), Rodrigo Abreu González (Ntra Sra de la Candelaria University Hospital), Rosa Borges Trujillo (Dr. Negrín University Hospital), Sybille Kaiser Giradot (Primary Care of Tenerife), Víctor Lorenzo Sellarés (University Hospital of Canary Island).
